# Pain Management in Pregnancy: Multimodal Approaches

**DOI:** 10.1155/2015/987483

**Published:** 2015-09-13

**Authors:** Shalini Shah, Esther T. Banh, Katharine Koury, Gaurav Bhatia, Roneeta Nandi, Padma Gulur

**Affiliations:** ^1^Department of Anesthesiology & Perioperative Care, University of California, Irvine, 333 The City Boulevard West, Suite 2150, Orange, CA 92868, USA; ^2^Department of Anesthesia, Critical Care and Pain Medicine, Massachusetts General Hospital, 55 Fruit Street, Gray-Bigelow 444, Boston, MA 02114, USA

## Abstract

Nonobstetrical causes of pain during pregnancy are very common and can be incapacitating if not treated appropriately. Recent reports in the literature show that a significant percentage of pregnant women are treated with opioids during pregnancy. To address common pain conditions that present during pregnancy and the available pharmacological and nonpharmacological treatment options, for each of the pain conditions identified, a search using MEDLINE, PubMed, Embase, and Cochrane databases was performed. The quality of the evidence was evaluated in the context of study design. This paper is a narrative summary of the results obtained from individual reviews. There were significant disparities in the studies in terms of design, research and methodology, and outcomes analyzed. There is reasonable evidence available for pharmacological approaches; however, these are also associated with adverse events. Evidence for nonpharmacological approaches is limited and hence their efficacy is unclear, although they do appear to be primarily safe. A multimodal approach using a combination of nonpharmacological and pharmacological options to treat these pain conditions is likely to have the most benefit while limiting risk. Research trials with sound methodology and analysis of outcome data are needed.

## 1. Introduction

Nonobstetrical causes of pain during pregnancy are very common and can be incapacitating if not treated appropriately. A recent study, with a cohort of more than 500,000 pregnant women in the United States, found that 14% of women filled a prescription for an opioid at least once during the antepartum period and 6% of women received opioids throughout all trimesters [1]. Additionally, another study reporting on more than one million pregnant women found that prescription opioids were dispensed to approximately one out of five women during pregnancy [2].

While these results reflect the high incidence of pain syndromes during pregnancy, they also highlight the increasing use of narcotics for pain management through the antepartum period. Given that between the years of 1999 and 2010 death from opioid pain relievers has increased fivefold in the United States, it is important to provide more multimodal balanced pain management strategies [3]. While there is increasing awareness and use of nonpharmacological approaches in the management of pain overall, the literature and discussion on their use in the pregnant patient with the unique consideration of mother and fetus are sparse. In this review, we discuss evidence for both pharmacological and nonpharmacological approaches in managing painful conditions during pregnancy. We present an approach to the diagnosis and treatment of some common painful conditions that may present during pregnancy.

## 2. Methodology

This narrative review incorporates a descriptive summary and integration of the available evidence on both pharmacological and nonpharmacological approaches to pain management during pregnancy. A literature search was conducted using medical databases including MEDLINE, PubMed, Embase, and Cochrane using the keywords “pain management,” “pregnancy pain,” “obstetric pain,” “opioid use,” and “non-pharmacological treatment” in our searches. We specifically examined papers discussing multimodal and nonpharmacological treatment options for pregnant women with nonobstetric pain. The resulting findings were then used to guide the discussion on the most safe and effective modalities of treatment.

## 3. Common Pain Presentations

Throughout pregnancy, several anatomic and physiologic changes occur in the body. These changes can precipitate pain, which in some cases can lead to disability. Additionally, a pregnant state can exacerbate preexisting painful conditions. Pain conditions during pregnancy may be further grouped into a systems-based classification such as musculoskeletal, rheumatologic, neuropathic, and pelvicoabdominal pain syndromes.

## 4. Musculoskeletal and Rheumatologic Pain

### 4.1. Low Back Pain

Low back pain is a common problem among pregnant women as it impacts approximately half of pregnancies. In fact, many obstetricians consider low back pain a normal finding in pregnancy [3]. Several factors have been associated with the development of low back pain during pregnancy. More specifically, laborious work, a history of low back pain before pregnancy, and a previous history of pregnancy-related low back pain have been identified as risk factors for the development of pregnancy-related low back pain. By and large, pain syndromes during pregnancy impact the abdominopelvic musculoskeletal system, thereby causing abnormal strain on the axial low back elements. Here, we present the most common lumbar pathologies during pregnancy and offer effective evidence based treatment strategies.

#### 4.1.1. Etiology

Normal changes of pregnancy include mechanical strain from the enlarging gravid uterus and ensuing adjusting lumbar lordosis. In addition, increasing weight gain and ligamentous laxity can together impede the neutral anatomical position. As a result, increased gravitational and mechanical load is placed upon the supportive lumbar discs, and increased strain is measured upon the paraspinal muscle beds. Interestingly, the incidence of a herniated nucleus pulposus (HNP) in pregnancy is 1 : 10,000, demonstrating that pregnant women do* not* have an increased prevalence of lumbar disc abnormalities as compared to the general population [4]. It appears that the radicular symptoms are secondary to compression neuropathy from the gravid uterus. In addition to discogenic pain, decreased ligamentous support to the sacroiliac joints can develop due to the influence of relaxin, a hormone secreted by the corpus luteum that softens collagen and loosens structural pelvic ligaments. Increased laxity can cause pain by the combination of an exaggerated range of movement in these joints and distention and increased mechanical strain. Low back pain can also be caused when degenerative spondylolisthesis is exacerbated by pregnancy in susceptible women [5].

#### 4.1.2. Evaluation

Diagnostic evaluation can be approached by a focused history and physical exam, which often assists the clinician to rule out other causes of low back discomfort such as pyelonephritis and renal calculi. A brief understanding of the pathophysiology of axial low back pain aids in rapid diagnosis. While the majority of pregnancy related back pain is myofascial in origin, we present here some diagnostic pearls to assist in evaluation of more complex presentations. For example, presentation of a patient with lumbar paraspinal muscle bed tenderness alleviated with massage and heat suggests myofascial pain. Alternatively, presentation of a patient with low back pain exacerbated in the upright position (with gravitational load) alleviated with recumbence associated with radiation of pain following a dermatomal pattern (radiculopathy) in the lower extremities is highly suggestive of a herniated nucleus pulposus and lumbar nerve root compression. Positive straight leg test (reproducible low back pain with radicular symptoms) and/or loss of unilateral deep tendon reflex are consistent with HNP. Careful examination of the sacroiliac joints should also be made. A patient with unilateral low back pain well localized with one fingerbreadth to low lateral spine suggests sacroiliitis, likely secondary to ligamentous laxity of the superior portion of the joint. Routinely, further diagnostic workup, such as imaging, is not commonly indicated as most diagnoses can be made based on well history and physical examination alone, and radiation exposure to the fetus should be limited. The exception would be in the case of presenting motor or sensory deficits, or presentation of cauda equina syndrome, in which an MRI may aid in rapid diagnosis and urgent treatment. Although MRI appears to be safe during pregnancy, there are no long-term studies examining the safety of fetal exposure to intense magnetic fields to date [4].

#### 4.1.3. Management

Most therapeutic strategies encourage preventive measures among pregnant women and those who are planning to become pregnant. It has been shown that women who participate in prophylactic education and strengthening programs during early pregnancy can avoid problems from low back pain [6]. There is also evidence showing that women who are in good physical shape before pregnancy experience less back pain during pregnancy [7].

*Postural Techniques and Physical Therapy*. When treatment is necessary for low back pain, conservative management is the ideal option. Treatment starts with education and activity adjustments. Educational strategies focus on back care measures, such as ergonomics, which teaches women correct posture; pregnant women learn how to stand, walk, or bend properly, without causing stress on the spine. Accurate posture is essential to improve low back pain. Braces that ensure correct body posture are also available if the instructions are not enough. In regard to activity modifications, scheduled rest during the day is helpful for relieving muscle spasms and acute pain. During this time, posture is again important as both feet should be elevated, which will help flex the hips and decrease the lumbar lordosis of the spine [5]. Evidence has shown that most patients responded positively to activity and postural modifications [5].

If pregnancy-related low back pain is not relieved through prophylactic education and activity modifications, then physical therapy may be valuable. Studies have found that pregnant women with low back pain, who participate in both education and physical therapy, have less pain and disability, higher quality of life, and improvement on physical tests [5]. Physical therapy encompasses several factors such as postural modifications, back strengthening, stretching, and self-mobilization techniques. Functional stability can be maintained throughout pregnancy by strengthening the muscles around the lumbar spine through various back exercises. Specifically, flexion exercises help make the abdominal muscles stronger and decrease the lumbar lordosis, whereas extension exercises help increase paraspinal muscles strength [8]. Through physical therapy, pregnant women may increase muscle strength and thereby lessen symptoms of low back pain. Physical therapy exercises including pelvic tilt, knee pull, straight leg raising, curl up, lateral straight leg raising, and the Kegel exercises have been identified as particularly efficacious in relieving lumbar pain [6].

*Complementary and Alternative Medical Treatments*. Other nonpharmacologic treatment modalities that have been studied during pregnancy include acupuncture, manual therapy, water therapy, transcutaneous nerve stimulation, stabilization belts, yoga, and other complementary and alternative medical treatments. Acupuncture is generally considered safe during pregnancy [9, 10]. It is believed that acupuncture works by stimulating the body's own pain relieving opioid mechanisms [9]. No significant adverse effects have been found [5, 6], although it is recommended to avoid acupuncture points that can stimulate the cervix and uterus, as they can induce labor [9, 10]. Studies have compared the efficacy of acupuncture compared to physical therapy for the treatment of low back pain, and the results highlight the superiority of acupuncture for relieving pain and decreasing disability [9]. Manual therapy is also important for decreasing pain as it influences the spinal “gating” mechanism as well as the pain suppression system [9]. One form of manual therapy is osteopathic manipulative treatment (OMT), which is offered by osteopathic physicians. One randomized controlled trial studied low back pain and related symptoms in the third trimester of pregnancy by comparing OMT and standard obstetric care, standard obstetric care and sham ultrasound treatment, and standard obstetric care alone. The results showed that, during the third trimester of pregnancy, OMT reduces or stops the deterioration of back pain related functioning [11]. Another notable treatment modality is water therapy, which involves physical exercises in a pool. Studies have shown the benefits that water therapy has to ameliorate pain and to lessen the demand for sick leave in women with pregnancy-related low back pain [5, 6, 12]. On the other hand, there is limited data that transcutaneous electrical nerve stimulation (TENS) is an efficacious and innocuous treatment modality for low back pain during pregnancy. One study compared TENS to exercise and acetaminophen and found that TENS improved low back pain more effectively; however, Keskin and colleagues suggested that further studies are needed before generalizing these results [13]. The TENS unit is used for labor analgesia in many countries and often used for pain management during pregnancy. It is recommended to keep the current density low and avoid certain acupressure points [14]. Please refer to Table 3 for more information on complementary and alternative medicine options.

*Medication Management*. In 1979, the Food and Drug Administration (FDA) in the United States adopted a five-category labeling system for all drugs approved in the US which rates the potential risk for teratogenicity to the fetus based on available scientific evidence (see Table 1). However, our present knowledge about risks of pain medication use during pregnancy is incomplete and the physician must clinically weigh the risks and benefits of effectively managing pain against embryonic malformations individually.

The underlying principle in management of any parturient population is to minimize the use of medications considering any potential harm to the developing fetus during the course of pregnancy. One of the major limitations in evaluating fetal-drug interactions is understanding the* degree* to which congenital or teratogenic effects may occur, not only causation. An example of this concept is the drug class of anticonvulsants, in which mental retardation has been reported; however the degree to which it occurred in offspring reveals no consistent conclusions [19].

Pregnant women experiencing incessant pain requiring pharmacologic treatment should use acetaminophen as a first line drug. Acetaminophen (category B) provides similar analgesia as nonsteroidal medications without the antiprostaglandin or platelet inhibition effects of NSAIDS. Antiprostaglandins, such as aspirin and nonsteroidal anti-inflammatory drugs (NSAIDs), are typically contraindicated throughout pregnancy, and if they are used at all, it should only be in the first and second trimester, as these medications hold the risk of causing premature closure of the ductus arteriosus in the fetus if administrated at near term, as well as vasoconstriction of the uterine arteries during the third semester [5, 6]. They are labeled as category C classification. For neuropathic pain states, anticonvulsants such as gabapentin or antidepressants such as amitriptyline are routinely prescribed to the general population to aid in abating paresthesias or radiculopathies. However, there is very little scientific data about the safety of gabapentin in pregnant women or fetuses, although review of evidence from epileptic pregnant patients is highly associated with neural tube defects, mental deficiency, and craniofacial abnormalities [4]. Gabapentin has not been categorized as yet within the FDA Pregnancy Risk Classification System. Antidepressants are labeled with category D classification.

For unrelenting severe pain, pregnant women may receive opioid medications, although the prescribing physician must be cautious with the medication regimen to avoid opioid withdrawal in the newborn. Most of our scientific knowledge on opioid use during pregnancy has been extracted from large observational studies of opioid dependent and abusing mothers [40]. As such, there is no evidence to suggest a relationship between exposure to any of the opioid agonists during pregnancy and frequently occurring large categories of major or minor malformations in the fetus. Data from large surveillance studies have pointed to possible associations for individual defects, but the incidence is not statistically greater than the general population, though independent confirmation is needed [48]. Most opioids are labeled as category B classification, along with acetaminophen, with the exception of codeine (category C). Please refer to Table 2 for more information about specific drugs and indications in each trimester.

*Interventional Options and Evidence*. Expert consultation in management of patients with severe uncontrolled pain in the context of safety to the parturient and fetus should be considered. Firstly, patient expectations of the amount of relief should be managed to understand that complete relief may not be possible until weeks after the delivery of the fetus as the body returns to anatomical posturing. When preventative and medication measures fail, many options and strategies exist to improve patient symptomology and functioning using an interventional approach.

Options for low back pain management refractory to conservative management may include an interventional option. The strongest evidence of the efficacy of epidural steroids seems to be in patients with symptoms attributable to disc pathology presenting acutely with signs of radiculopathy [49]. In such patients we feel it is reasonable to proceed with epidural steroid placement before obtaining imaging studies. Fluoroscopy-guided injections of steroids or local anesthetics require exposure to ionizing radiation; therefore, options that could potentially be used during pregnancy include blind injections, MRI-guided injections, and ultrasound-guided injections, although image-guided procedures have a significantly greater margin of safety and should be utilized when feasible [10, 50]. Rosenberg and colleagues conducted a prospective, double-blind, correlational outcome study on the use of blind injections, which demonstrated that blind injections are typically not successful and that image-guided procedures are preferred [51]. On the other hand, ultrasound-guided epidural injections have been described as very precise [10, 50], although the success of the procedure can be contingent on the skill and experience of the operator [10]. For radicular pain, ultrasound-guided selective nerve root blocks are superior to a caudal approach, and for sacroiliitis, ultrasound guided sacroiliac joint injections are an excellent option.

#### 4.1.4. Prognosis

Within a few months after delivery, most women experience improvement of their pain symptoms [6]; however, some women continue having residual pain [6]. Specifically, in a prospective, 3-year follow-up study, about 20% of women reported that they continued to experience pain after pregnancy [56].

### 4.2. Joint Pain

Healthy women can present with diverse musculoskeletal changes during pregnancy, such as joint pain. These symptoms usually raise suspicion of inflammatory diseases like systemic lupus erythematous (SLE) or rheumatoid arthritis. However, developing new-onset rheumatoid arthritis during pregnancy is rare, and some studies even suggest that pregnancy is protective against new-onset rheumatoid disease [57]. Choi and colleagues showed arthralgia usually presented in the third trimester, with the proximal interphalangeal joint of the hand being the most commonly involved [58]. The prognosis was generally good, with most cases improving spontaneously. Women with preexisting inflammatory disease often experience altered disease activity during pregnancy. It is expected that, in pregnant women with SLE, there is increase in disease activity, whereas in pregnant women with rheumatoid arthritis there is a decrease in disease activity [58, 59].

#### 4.2.1. Etiology

Normal physiologic changes seen in pregnancy, such as soft tissue swelling and joint laxity, are also thought to be predispositions to joint pain. Hormonal changes throughout pregnancy, including increasing estrogen, progesterone, relaxin, and cortisol levels, have also been associated with these joint symptoms, resulting in pain and stiffness, and sometimes arthralgia [58].

#### 4.2.2. Management

The current literature on the management of joint pain in previously healthy pregnant women is limited. Presently, most of the available studies focus on managing joint pain in women with preexisting rheumatologic conditions [60]. A multidisciplinary treatment plan directed by a rheumatologist is suggested for pregnant women with rheumatologic disease. Use of complementary and alternative medicine options may be of some value for these patients as detailed in Table 3.

#### 4.2.3. Prognosis

During the postpartum period, there has been an escalation in the onset of new rheumatoid arthritis [61]. New onset of polyarthralgia or polyarthritis in the postpartum should raise the suspicion for this diagnosis.

### 4.3. Neuropathic Pain—Carpal Tunnel Syndrome (CTS)

Neuropathic pain can be produced by common conditions including carpal tunnel syndrome and meralgia paresthetica.

#### 4.3.1. Etiology

The prevalence of CTS ranges from 2.3% to 35% in pregnant women [62]. Hormonal changes associated with pregnancy and associated tissue edema have both been implicated [63, 64]. Electrophysiological changes in median nerve function have also been reported in asymptomatic pregnant women [65].

#### 4.3.2. Management

For the management of CTS, both activity modification and the use of splints in neutral position throughout the night have shown some success. Activity modification includes eschewing extreme flexion and/or extension and avoiding extended exposure to vibration. Thermoplastic night splints have been helpful, as several women have experienced symptom relief after two weeks of use [66]. Physical therapy and NSAIDs have also been recommended for CTS. If CTS symptoms persist, therapy directed specifically towards edema reduction is suggested. Additionally, steroid injections have been suggested as they have been shown to offer relief in up to 80% of patients. In extreme cases, surgical decompression may be necessary.

#### 4.3.3. Prognosis

Symptoms of CTS typically resolve after delivery. However, one study found that although women's symptoms had improved, the median nerve's distal sensory conduction velocity continued to be delayed in 84% of women one year after delivery [67].

### 4.4. Neuropathic Pain—Meralgia Paresthetica

#### 4.4.1. Etiology

Meralgia paresthetica is a sensory mononeuropathy that specifically involves the lateral femoral cutaneous nerve of the thigh. It transpires when the lateral femoral cutaneous nerve is compressed, as it is then forced to pass under the tensor fascia lata at the inguinal ligament. Patients typically describe an insidious onset of a painful burning sensation on the lateral aspect of the thigh, aggravated during side-sleeping and prolonged sitting or standing. Examination will reveal an exaggerated lumbar lordosis and sensory deficit on the lateral aspect of thigh with preserved motor function and reflexes.

#### 4.4.2. Management

Typically, treatment is usually not needed for meralgia paresthetica and symptoms abate with delivery of fetus. Stretching exercises such as the “cat-camel” position help alleviate pain temporarily. However, if symptoms persist, pain may be relieved by local infiltration of steroids and local anesthetics at the point of maximal tenderness or a lateral femoral cutaneous nerve block [62].

#### 4.4.3. Prognosis

Usually this condition is self-limited and resolves after delivery.

## 5. Pelvic and Abdominal Pain

### 5.1. Pregnancy-Related Pelvic Pain

#### 5.1.1. Etiology

The cause of pregnancy related pelvic pain is multifactorial. Pain seems to occur from increased motion in the pelvic girdle which is associated with increased ligamentous laxity, which occurs due to the influence of the hormones relaxin and estrogen [5, 68]. It has been proposed that there is a correlation between relaxin levels during pregnancy and pelvic pain. As a result of elevated relaxin concentrations, the symphysis pubis expands during the 10th to 12th weeks of pregnancy [5]. This widening can be painful, as it allows for increased mobility of the joints. This pain is exacerbated by exercise and sometimes mechanical strain. Risk factors for pregnancy-related pelvic pain include strenuous work, previous low back pain, previous history of pregnancy-related pelvic pain, and previous trauma to the pelvis [68].

#### 5.1.2. Management

Patient education is an important part of managing pregnancy-related pelvic pain. Information regarding the condition not only helps to reduce fear, but also encourages patients to become an active part in their treatment and rehabilitation. These patients should be provided with material about ergonomics and physical activity for their pain. Women with pregnancy-related pelvic pain should avoid maladaptive movements such as unequal weight bearing on legs, hip abduction, and activities that strain joints to their extreme.

Nonpharmacologic modalities recommended for the treatment of pelvic pain during pregnancy include massage, water gymnastics, acupuncture, pelvic belts, and exercise. Massage can be valuable as a part of a multidisciplinary treatment for pelvic pain during pregnancy, as it is not recommended as an individual treatment during this time. Aqua therapy has been suggested to mitigate pain and to decrease work absences [68]. As for acupuncture, studies suggest that it is advantageous as it alleviates pelvic pain without serious adverse effects during late pregnancy [68]. Pelvic belts can be used, but they should only be applied for short periods of time [68]. The role of exercise for reducing pelvic pain throughout pregnancy remains unclear. One study found that the risk of developing pregnancy related pelvic pain was decreased for women who spent more time becoming physically fit before pregnancy [69]. Additionally, women with pregnancy-related pelvic pain in the postpartum may receive some benefit through stabilizing exercises. However, a different study reported that exercises that help stabilize the pelvic area neither lessened the pain intensity nor reduced the recovery period after delivery [70]. Please refer to Table 3 for more information about complementary medicine options.

Acetaminophen is the drug of choice for pelvic pain during pregnancy. NSAIDs may offer superior pain relief; however, they must be used with caution and should be avoided in the third trimester. Please refer to Table 2 for more information about specific drugs and their use in each trimester.

#### 5.1.3. Prognosis

Pregnancy-related pelvic pain is typically described as a self-limiting condition, and symptoms usually resolve after delivery. One study showed that pain related to the pelvic joints throughout pregnancy could continue in 8.5% of women for at least two years after parturition [71].

### 5.2. Abdominal Nerve Entrapment—Anterior Cutaneous Nerve Entrapment Syndrome

#### 5.2.1. Etiology

Abdominal pain in pregnancy has various origins, and while most complaints involve the abdominal viscera, the abdominal wall itself can be a source of chronic pain in pregnancy. Anterior Cutaneous Nerve Entrapment Syndrome (ACNES) usually is reported after surgery, as the small cutaneous nerve fibers become entrapped in skin incisions. The syndrome was first reported in 1926 by Dr. Carnett, a Family Physician, in which he outlined nonvisceral pathology contributing to chronic abdominal pain. Pain is described as very well localized and lateral to the umbilicus. Physical examination will demonstrate a positive Carnett's sign (pain worsened with Valsalva maneuvers such as a crunch or sit up) and alleviated with rest. In pregnancy, the increasing abdominal wall size can stretch the cutaneous nerves and cause chronic pain. It has been proposed that the changes in the thoracic and abdominal wall from uterine growth can lead to entrapment of the anterior cutaneous nerves [72].

#### 5.2.2. Management

Ultrasound-guided transversus abdominis plane (TAP) blocks or rectus sheath blocks can be used to alleviate pain from Anterior Cutaneous Nerve Entrapment Syndrome. The choice of which block to use is indicated by the location of the pain, and these blocks may be considered for both diagnostic and therapeutic approaches.

#### 5.2.3. Prognosis

Upon appropriate diagnosis, patients respond very well to TAP blocks or rectus sheath blocks, usually requiring only a single injection [73, 74]. In most cases, a combination of stretching and intervention provides the best results. A case series reported on three pregnant women with disabling pain in the lower abdominal pain, all of which responded to local selective block to these nerves [72].

### 5.3. Intercostal Neuralgia

#### 5.3.1. Etiology

Intercostal neuralgia may arise as a result of lesions at the level of the spinal cord, nerve trunks, roots, or terminals. It has been suggested that the enlarging gravid uterus, which leads to mechanical stretch on the lower intercostal nerves, can cause intercostal neuralgia [75]. It has also been proposed that postural variations throughout pregnancy can assist in generating nerve root irritation when the nerves transverse the neural foramina [76]. Intercostal neuralgia may manifest with radicular pain in the distribution of a thoracic root or intercostal nerve [77]. Pain is described as a burning or radiating pain, associated with late gestation (increased uterine displacement), and occasionally followed by bouts of coughing, which can cause a secondary costochondritis.

#### 5.3.2. Management

In a review of the literature, topical lidocaine patches or creams, intercostal nerve blocks, and/or epidural steroid injections have evidentiary support for successful treatment for pregnant women with intercostal neuralgia [77].

#### 5.3.3. Prognosis

Pain usually remits after delivery once pressure on the nerve or root is relieved [77].

## 6. Conclusion

With an ever-increasing rate in the rise of parturient utilizing opioid pain medications, it is reasonable to assume that many obstetricians may be uncertain about adequate treatment options to offer their population. Evaluation as well as effective management is limited by the relative contraindication of radiography in the workup and the risks to the fetus associated with pharmacologic therapy against providing effective analgesia to the patient. Evidence on nonpharmacologic strategies, while limited, is of value. If pharmacologic therapy is required, the decision to use it must be based on the risks and benefits to the mother and the fetus. Management of these patients should be with a multidisciplinary team, providing all the therapeutic options to assure the well-being to the patient, minimize fetal teratogenicity, and avoid chronic symptoms and long-term disability. Nevertheless, an understanding of frequently occurring pain complaints along with quick diagnostic evaluation, risks of pain medications to the maternal-fetal unit, complementary alternative options, and expert consultation allows the obstetrician to easily help women achieve a more enjoyable and functional pregnancy.

## Figures and Tables

**Table 1:** Classification system for fetal risk. FDA classes: in 1979, the United States Food and Drug Administration (FDA) established a five-category classification system for fetal risk from exposure to certain class of medications.

FDA classification	Definition^a^	Examples
Category A	Controlled studies in women fail to demonstrate a risk to fetus. The possibility of harm to the fetus appears remote.	Multivitamins

Category B	Either animal studies have not demonstrated a fetal risk but there are no controlled human studies *or* animal studies have indicated an adverse effect that was not confirmed in controlled studies in women in the 1st trimester (and there is no evidence of risk in the later trimesters).	(i) PO acetaminophen (ii) Opioids: nalbuphine (iii) Local anesthetic: lidocaine

Category C	Teratogenic or embryocidal risk indicated in animal studies, but controlled studies in women have not been done *or* there are no controlled studies in animals or humans.	(i) NSAIDs: sulindac, naproxen (ii) Opioids: codeine, butorphanol, fentanyl, hydrocodone, levorphanol, methadone, morphine, oxycodone, and oxymorphone (iii) Antidepressants: fluoxetine (iv) Tricyclic antidepressants: amitriptyline, imipramine (v) Anticonvulsant: gabapentin (vi) Drugs used for migraine: metoprolol, propranolol, sumatriptan, nifedipine, and verapamil

Category D	Positive evidence of fetal risk, but use in pregnant woman is acceptable since the maternal benefit outweighs the risk to the fetus.	(i) NSAIDs: aspirin (ii) Steroids: cortisone (iii) Anticonvulsants: diazepam, phenobarbital, and phenytoin

Category X	Animal and human studies demonstrate fetal abnormalities *or* there is evidence of fetal risk based on human experience *or* both; the risk outweighs any possible benefit. The drug is *contraindicated* in women who are or may become pregnant.	(i) Antimigraine: ergotamine (ii) Antidepressants: paroxetine (iii) Anticonvulsants: valproic acid

^a^The definitions for the factors are derived from those used by the FDA [15].

In this classification system, all drugs are assigned to risk factor category A, B, C, D, or X based on scientific or clinical evidence of risk to the fetus. They do not refer to breastfeeding risk. A drug is reasonably safe when administered to a pregnant patient if labeled with category A. The FDA classification, however, does not assign any of the pain medications (nonsteroidal anti-inflammatory drugs, opioids, local anesthetics, steroids, tricyclic antidepressants, or antiepileptics) to category A [16, 17].

Despite the lack of literature on the safety of drugs in pregnancy and during lactation, the statistics show that drug use, over the counter and prescription, during pregnancy is widespread. A study published in 2004 found that almost one half of pregnant women received prescription drugs from FDA risk category C, D, or X [18].

**Table 2:** Specific drugs and indications in each trimester.

Drug	First trimester (weeks 1–12)	Second trimester (weeks 13–28)	Third trimester (weeks 28–40)	Labor	Postpartum and lactation
Acetaminophen Risk factor: B when taken orally (C for intravenous use) *650 mg every 4–6 hours or 1 g every 6 hours.*	Use with caution Strong evidence against increased risk of miscarriage [20], serious birth defects [21], IQ, or physical growth [22]. Associated with small increased risk of cryptorchidism in boys [23] and childhood asthma [24].	Use with caution Associated with small increased risk of cryptorchidism in boys [23] and childhood asthma [24].	Use with caution Associated with increased risk of childhood asthma [24].	Safe to use No increased risk of hemorrhage if the drug is given to the mother at term in standard doses [16].	Safe to use The American Academy of Pediatrics (AAP) considers acetaminophen to usually be safe during lactation [25]. Weak association with early infant exposure (first 6 months) with increased risk of childhood asthma [26]; more research is required.

*Nonsteroidal anti-inflammatory drugs (NSAIDs)* See individual drugs in the class for more detailed information, as risks vary per drug.	Studies are mixed on prenatal and early pregnancy use of NSAIDs and risk of miscarriage. Nakhai-Pour et al. [27] showed the association with NSAIDs as a class; however, Edwards and colleagues [28] did not find this association. A more recent study, with more than 65,000 women also did not find an increased risk of spontaneous abortion following exposure to NSAIDs [29]. One prospective study in pregnant patients with inflammatory rheumatic disease did not show a significant association with major birth defects nor harmful long-term effects caused by intrauterine exposure to these drugs when taken early to mid-pregnancy [30]. On the other hand, Ericson and Källén observed an increase in cardiac malformations in women with rheumatic disease exposed to NSAIDs in the first trimester [31]. There was no drug specificity for cardiac defects.	One prospective study in pregnant patients with inflammatory rheumatic disease did not show a significant association with major birth defects nor harmful long-term effects caused by intrauterine exposure to these drugs when taken early to mid-pregnancy [30].	Do not use. NSAIDs are generally linked to premature closure of the ductus arteriosus when taken in the third trimester of pregnancy, which in some cases may result in primary pulmonary hypertension of the newborn [16]. Large doses taken by mothers in the week before delivery can increase risk of intracranial hemorrhage in premature neonates [16].	The use of NSAIDs as tocolytics has been associated with an increased risk of neonatal complications, such as patient ductus arteriosus necrotizing enterocolitis and intraventricular hemorrhage [16].	NSAIDs in general seem to be safe during breastfeeding [16].

(i) Aspirin Risk factor: D *60–100 mg daily is generally not associated with adverse outcomes.*	Use only if clearly indicated Three studies including 11,000 NSAID-exposed pregnancies did not find a significant increase in the frequency of congenital malformations, nor was there an effect upon infant survival compared with unexposed pregnancies [32, 33]. Low-dose aspirin therapy (81 mg/day) is generally free of maternal or neonatal complications [34]. Weak evidence for increased associations with gastroschisis [35] and IQ/attention decrements in children [22].	Use only if clearly indicated In one study, aspirin was dose-dependently associated with congenital cryptorchidism, particularly during the second trimester [36].	Do not use, especially if there is increased risk of premature delivery The use of high-dose aspirin close to delivery has been shown to increase the incidence of clotting abnormalities, in addition to neonatal and perinatal bleeding such as hemorrhage in the CNS in the newborn [16]. Severe neonatal bleeding has been reported after premature delivery [37]. Premature closure of the ductus arteriosus can result when using a full-dose aspirin in this period. Persistent pulmonary hypertension of the newborn (PPHN) is a potential complication of this closure.	Use only if clearly indicated	Use only if clearly indicated Data has suggested that low dose aspirin, 81 mg/day, is generally considered safe; however, aspirin should be used with caution. Doses above 150 mg are contraindicated.

(ii) Ibuprofen Risk factor: C (prior to 28 weeks of gestation)/D (≥28 weeks of gestation) *400 mg every 4–6 hours as needed. *	Use with caution	Use with caution	Do not use Linked to premature closure of the ductus arteriosus, resulting in persistent pulmonary hypertension of the newborn (PPHN) [16].	Use with caution	Safe for breastfeeding women to use. AAP classifies ibuprofen as usually compatible with breastfeeding [25].

(iii) Ketorolac Risk factor: C (prior to 28 weeks of gestation)/D (≥28 weeks of gestation) *Single IV dose: 30 mg. Weight <50 kg: 15 mg. *	Use with caution	Use with caution	Do not use Linked to premature closure of the ductus arteriosus, resulting in persistent pulmonary hypertension of the newborn (PPHN) [16].	Use with caution	Use with caution AAP classifies ketorolac as usually compatible with breastfeeding [25].

(iv) Naproxen Risk factor: C *500 mg every 12 hours.*	Use with caution One study found an association between naproxen use and orofacial clefts [38]. However, the risk for these defects appears to be small [16].	Use with caution	Do not use Linked to premature closure of the ductus arteriosus, resulting in persistent pulmonary hypertension of the newborn (PPHN) [16].	Use with caution	Safe for breastfeeding women to use. AAP classifies naproxen as usually compatible with breastfeeding [25].

(v) Celecoxib Risk factor: C (prior to 28 weeks of gestation)/D (≥28 weeks of gestation). *200 mg twice daily.*	Use with caution	Use with caution	Do not use Linked to premature closure of the ductus arteriosus, resulting in persistent pulmonary hypertension of the newborn (PPHN) [16].	Use with caution	Use only if clearly indicated There is inadequate evidence to fully determine infant risk. Should only be used if the possible benefit outweighs the possible risk.

*Opioids* See individual drugs in the class for more detailed information, as risks vary per drug.	Use with caution In general, short-term, episodic use of opiates appears to be safe in pregnancy [16]. Few studies have evaluated opioid teratogenicity in the first trimester. Overall opinion is that there is minimal risk [39]. However, one study [40] showed an association with congenital heart defects, spina bifida, and gastroschisis. This study is limited by recall-bias. Opioid abuse and use of chronic opioids during pregnancy is associated with neonatal abstinence syndrome (NAS) [41].	Use with caution In general, short-term, episodic use of opiates appears to be safe in pregnancy [16]. Opioid abuse as well as use of chronic opioids during pregnancy is associated with neonatal abstinence syndrome (NAS) [41].	Use with caution In general, short-term, episodic use of opiates appears to be safe in pregnancy [16]. Opioid abuse as well as use of chronic opioids during pregnancy is associated with neonatal abstinence syndrome (NAS) [41]. Onset of withdrawal signs is sooner in infants exposed to opioids with shorter half-lives, such as morphine and oxycodone.	Use with caution Maternal opioids pass readily into fetal circulation and can cause fetal respiratory depression. Opioids should be avoided when delivery of a premature neonate is expected.	Use with caution The short-term use of opiates during breastfeeding appears to be safe. Infants should be closely monitored for signs of respiratory depression [16, 25].

(i) Morphine Risk factor: C *15 mg, 30 mg tabs; 10 mg, 20 mg/5 mL elixir*	Use with caution No reports linking the therapeutic use of morphine with major congenital defects have been reported [16].	Use with caution Onset of withdrawal signs is sooner in infants exposed to opioids with shorter half-lives, such as morphine.	Use with caution Onset of withdrawal signs is sooner in infants exposed to opioids with shorter half-lives, such as morphine.	Use with caution	Use with caution AAP classifies morphine as usually compatible with breastfeeding [25].

(ii) Fentanyl Risk factor: C *Intramuscular, intravenous, intrabuccal, transdermal, or epidural*	Use with caution	Use with caution	Use with caution	Use with caution	Use with caution AAP classifies fentanyl as usually compatible with breastfeeding [25].

(iii) Hydrocodone Risk factor: C	Use with caution	Use with caution	Use with caution	Use with caution	Use with caution Appears to be safe in breastfeeding as very little hydrocodone is transferred to the milk [42]. However, there is inadequate evidence to fully determine infant risk. Should only be used if the possible benefit outweighs the possible risk.

(iv) Codeine Risk factor: C	Use with caution Birth defects (including some heart defects) have been reported with maternal use of codeine in the first trimester of pregnancies [16, 40]. However, in another study, no effects were observed on infant survival or congenital malformation rate [33].	Use with caution	Use with caution	Use with caution The use of codeine during labor may produce neonatal respiratory depression [16].	Use with caution. AAP has classified codeine as usually compatible with breastfeeding [16]. However, toxicity has been reported [43]. The recommendation is to avoid long-term consumption of codeine-containing products during breastfeeding. Short-term therapy, such as 1-2 days, with close monitoring of the infant for symptoms of opioid toxicity is recommended [16].

(v) Methadone Risk factor: C *Oral, subcutaneous, intramuscular, or intravenous*	Use with caution Methadone has been shown to have a favorable risk/benefit ratio if the user is a part of a comprehensive opioid dependence maintenance program during pregnancy. Methadone-maintenance has been associated with longer gestation and increased birth weights in comparison to nonmaintenance controls [44]. If maintenance medication is necessary, treatment should begin with the lowest effective dose [45].	Use with caution Clearance of methadone increases during the second and third trimester, which may cause withdrawal symptoms and necessitate dose adjustment [46]. Onset of withdrawal signs is longer in infants exposed to opioids like methadone.	Use with caution Clearance of methadone increases during the second and third trimester, which may cause withdrawal symptoms and necessitate dose adjustment [46]. Onset of withdrawal signs is longer in infants exposed to opioids like methadone.	Use with caution	Use with caution Breastfeeding is likely safe, based on studies that show transfer to milk is extremely low [16, 47]. AAP classifies methadone as usually compatible with breastfeeding [25].

**Table 3:** Complementary medicine.

Therapy	1st trimester	2nd trimester	3rd trimester	Labor	Postpartum
CAM (acupuncture, acupressure, massage) [52, 53]	Do not use (may stimulate uterine contractions)	Use with caution (experienced therapist; not a high risk pregnancy)	Use with caution (experienced therapist; not a high risk pregnancy)	Use with caution (experienced therapist; not a high risk pregnancy)	Safe

Physical therapy (TENS unit) [52]	Safe	Safe	Safe	N/A	Safe

Hydrotherapy/aqua therapy [54]	Use with caution (avoid hot tubs)	Use with caution (avoid hot tubs)	Use with caution (avoid hot tubs)	Use with caution (birthing pool)	Safe (avoid if C-section)

Cognitive behavioral therapy, biofeedback [52]	Safe	Safe	Safe	Safe	Safe

Chiropractic care [54]	Use with caution (pressure off abdomen)	Safe	Use with caution (avoid lying on back)	N/A	Safe

The use of complementary and alternative medicine (CAM) is on the rise in Western countries as the research focusing on its use has intensified over the last decade [55]. The World Health Organization (WHO) defined CAM as “a broad set of health practices that are not part of a country's own tradition, or not integrated into its dominant health care system [52].” CAM is utilized in various treatment populations including parturient. There have been several large-scale surveys, which indicate that 48% of all women of childbearing age currently use at least one CAM therapy for health-related problems [52]. Studies have shown that women, who are older, have higher education and income and are more likely to use CAM therapies for their physical symptoms during pregnancy. Other associations such as previous use of CAM, primiparity, nonsmoking, and planning a natural birth were also directly correlated with consumption of CAM [53]. A common belief amongst users of CAM is that it is considered natural, safe, and/or having equal efficacy when compared to medical treatments for pregnancy and its related symptoms. However, the research to support the common beliefs and perceptions is limited and the potential risks to mother and fetus are unknown [55].
